# Effect of ^18^F-FDG PET/CT Imaging Combined with 3D-Printed Templates on Biopsy Efficacy in Patients with Lung Tumor

**DOI:** 10.12669/pjms.41.4.9828

**Published:** 2025-04

**Authors:** Jianyang Zhang, Cheng Ran, Jing Chen, Huimin Song, Xianbo Li

**Affiliations:** 1 Jianyang Zhang, Nuclear Medicine Department, Baoding NO.1 Central Hospital, Baoding 071000, Hebei, China; 2 Cheng Ran, E.N.T. Department, Affiliated Hospital of Hebei University, Baoding 071000, Hebei, China; 3 Jing Chen, Nuclear Medicine Department, Baoding NO.1 Central Hospital, Baoding 071000, Hebei, China; 4 Huimin Song, Nuclear Medicine Department, Baoding NO.1 Central Hospital, Baoding 071000, Hebei, China; 5 Xianbo Li, Thoracic Surgery Department, Baoding NO.1 Central Hospital, Baoding 071000, Hebei, China

**Keywords:** ^18^F-FDG, PET/CT, 3D-Printed Templates, Percutaneous Aspiration Biopsy, Lung, Diagnostic Efficacy

## Abstract

**Objective:**

To investigate the effect of ^18^F-FDG PET/CT imaging combined with 3D-printed templates on the biopsy results and diagnostic efficacy in patients with lung tumors.

**Methods:**

This was a comparative study. A total of 80 patients with suspected lung tumors admitted to Baoding NO.1 Central Hospital from May 2022 to December 2023 were selected as the research subjects and divided into the study group and the control group per different detection methods. Patients in the control group were treated with ^18^F-FDG PET/CT imaging-guided percutaneous aspiration biopsy of the lung, while those in the observation group underwent ^18^F-FDG PET/CT imaging-guided percutaneous aspiration biopsy of the lung combined with 3D printed templates. Afterward, the biopsy results of the two groups and their diagnostic efficacy and complications were compared.

**Results:**

No significant differences were observed in tissue length and scanning time between the two groups (P>0.05). The operation time and number of biopsies in the observation group were less than those in the control group, with significant differences (P<0.05). Meanwhile, there was no statistically significant difference in specificity (15.00% vs. 27.50%) and misdiagnosis rate (2.50% vs. 10.00%) between the two groups (P>0.05). Moreover, the incidence of complications in the observation group was 5.00%, lower than the control group (7.50%), with no statistically significant differences (*χ²*=0.213, P=0.644).

**Conclusion:**

The observation group was found to have superior effect than control group, with fewer punctures and shorter puncture time, making it worthy of promotion in clinical application.

## INTRODUCTION

Lung tumors, especially lung cancer, are currently one of the most prevalent malignancies worldwide, with the highest incidence and mortality rates, and their incidence is increasing year by year.^1^ Since the treatment outcome is subject to the pathological findings of patients when it comes to treating lung tumors, early diagnosis of the nature of lung tumors is crucial for selecting subsequent treatment options. However, most early lung tumors often have no obvious clinical symptoms, and their imaging features are atypical, making them prone to misdiagnosis or missed diagnosis.^2^,^3^ Currently, clinical diagnostic methods for lung tumors include imaging and laboratory tests, fiberoptic bronchoscopy/lung biopsy, and thoracoscopy surgery, while pathological examination of lung tumor tissues remains the gold standard for diagnosing the nature of lung tumor lesions.^4^

At present, percutaneous lung biopsy is widely used in China for diagnosing lung tumor lesions, with excellent diagnostic accuracy and a low incidence of complications, which has been widely recognized and applied domestically.^5^ In regard to imaging, lung tumors mainly rely on computed tomography (CT) and positron emission tomography/computed tomography (PET/CT), in which PET/CT, as a non-invasive functional imaging method, can detect early metabolic changes in malignant tumors, enabling early detection and diagnosis of cancer. Fluorodeoxyglucose-F18 (^18^F-FDG) is the most commonly used imaging agent that detects functional abnormalities before significant morphological changes occur, which reflects the glucose metabolism status of lesions at the molecular level and also exhibits lesion morphology characteristics, thus playing an important role in the diagnosis, staging, and evaluation of efficacy for lung tumors.

Meanwhile, as a mature and widely used manufacturing technology with high fidelity, precision, and repeatability, 3D printing technology has been widely applied in the medical field.^6^ Currently, there are few reports on the application of ^18^F-FDG PET/CT imaging combined with 3D-printed templates for lung tumor biopsies.^7^ In this study, a comparative study was conducted on clinical data regarding the application of ^18^F-FDG PET/CT imaging combined with 3D-printed templates for lung tumor biopsies in Baoding NO.1 Central Hospital, to investigate the effect of 18F-FDG PET/CT imaging combined with 3D-printed templates on the biopsy results and diagnostic efficacy in patients with lung tumors.

## METHODS

A comparative analysis was conducted on the clinical data of patients with pulmonary nodules admitted to Baoding NO.1 Central Hospital from May 2022 to December 2023. Eighty patients were included per the inclusion and exclusion criteria and divided into the observation group (n=40) and the control group (n=40) by different puncture methods.

### Ethical Approval:

The study was approved by the Institutional Ethics Committee of Baoding NO.1 Central Hospital (No.:2023075; Date: October 23, 2023), and written informed consent was obtained from all participants.

### Inclusion criteria:

- Patients with complete clinical data, aged 18 to 70 years old.
- CT showed that the maximum diameter of pulmonary nodules ≤ 1 cm.
- No inflammation, atelectasis, regional lymphadenopathy, and other manifestations around the nodules.
- Patients who met the surgical indications and had puncture path.
- Patients and their families were informed of the study content and signed informed consent forms.

### Exclusion criteria:

- Patients without complete clinical data.
- Lesions adjacent to the great vessels.
- Patients complicated with heart, liver, kidney dysfunction, or other malignant tumors.
- Patients intolerant to surgery, puncture and with contraindications.
- Patients with complicated asthma, severe emphysema and bullae and other respiratory diseases.
- Patients with severe coagulation dysfunction and bleeding related diseases.

Patients in the control group fasted for six-hour one week before biopsy, with a blood glucose concentration of <10 mmol/L, and then were intravenously injected with ^18^F-FDG at 5.55~7.40 MBq/kg, followed by a whole-body scan using PET/CT (United Imaging) after resting quietly for 60 m. Afterward, PET/CT tomographic image reconstruction was performed using an iterative method, and a standard uptake value (SUVmax) >2.5 was used as the criterion for a positive high metabolic area. Following the fused PET/CT images, the nuclear medicine department and the biopsy physician selected the site with the maximum SUVmax >2.5 or the most metabolically active part as the biopsy target. An 18G coaxial biopsy gun was used, and the optimal body surface insertion point was marked using a grid marking method while avoiding vital organs, blood vessels, and ribs. After disinfection and anesthesia, the mass parenchyma was selected as the biopsy target, and the lesion tissue was sampled using CT localization-guided biopsy.

For the observation group, patients underwent biopsy using ^18^F-FDG PET/CT imaging combined with 3D-printed templates. Firstly, the chest CT data of patients was imported into the Image Processing System (United Imaging) for template modeling. The puncture point was determined based on the specific condition of the patients, and the puncture needle insertion angle was simulated and designed. After modeling, the information was imported into the 3D system to create the 3D-printed template. Subsequently, the PET/CT examination was performed as in the control group. After anesthesia and disinfection, the 3D-printed template was often placed at the fixed point of the chest wall.

The puncture point was rechecked, and the guiding needle was inserted along the “needle insertion tunnel” preset in the 3D-printed template. Once the puncture needle was inserted, the anchoring positioning needle was released, with the positioning wire guided into the chest cavity. After completing the positioning, the lesion tissue was sampled by biopsy using an 18G coaxial biopsy gun. Afterward, exfoliative cytology was performed on the obtained biopsy tissues from both groups first, followed by pathological examination. After the biopsy, a chest CT scan was performed again to confirm the absence of pneumothorax and bleeding spots. Close attention should be paid to the patients’ vital signs for 6-8 hour, along with observation of the occurrence of complications, so as to provide timely symptomatic treatment if needed. All operations were performed by the same group of doctors.

### Outcome Indicators:

- ***Biopsy results:*** Tissue length, number of biopsies, scanning time, and operation time.
- Comparison of the examination results between the two groups using the pathological results of surgical resection as the “gold standard”.
- Comparison of the accuracy, sensitivity, specificity, missed diagnosis rate, and misdiagnosis rate of puncture results between the two groups.
- Puncture complications, including pneumothorax, bleeding, hemoptysis, etc.

### Statistical Analysis:

SPSS 23.0 software was used for analysis. Measurement data were expressed as mean ±standard deviation(*χ̅*±*S*), with the independent sample t-test used for inter-group comparison; count data were expressed as number of cases and percentage (%), and the χ^2^ test was utilized for inter-group comparison. P< 0.05 was considered statistically significant.

## RESULTS

The observation group consisted of 25 males and 15 females, aged 42-70 years, with an average age of (58.88±6.79) years. Meanwhile, there were 23 males and 17 females in the control group, aged 44-70 years, with an average age of (58.10 ± 7.82) years. All patients were followed up. No significant difference was observed in the general profile between the two groups (P > 0.05) (Table-I).

**Table-I:** Comparison of General Profile between the two groups.

Group	n	Gender(n)		Age(years)	Lesion Diameter(cm)	BMI(kg/m^2^)
		M	F			
Observation	40	25	15	58.88±6.79	3.25±0.61	24.32±4.34
Control	40	23	17	58.10±7.82	3.08±0.78	24.09±3.54
*t/χ²*		0.208		0.473	1.079	0.257
*P*		0.648		0.637	0.284	0.798

There was no significant difference in tissue length and scanning time between the two groups (P > 0.05). The operation time and number of biopsies in the observation group were less than those in the control group, with significant differences (P < 0.05) (Table-II).

**Table-II:** Comparison of Biopsy Indicators between the two groups (*χ̅*±*S*).

Group	n	Lesion Length (mm)	Scanning Time (s)	Operation Time (s)	Number of Biopsies (times)
Observation	40	11.57±3.70	6.85±1.14	12.85±1.93	3.73±0.88
Control	40	11.02±3.63	7.03±1.21	14.43±1.75	4.18±0.93
*t*		0.677	0.665	3.822	2.226
*P*		0.500	0.508	0.000	0.029

In the observation group, 33 and 7 cases were respectively confirmed positive and negative by surgical results, and 31 and 9 cases were respectively positive and negative by needle biopsy. In the control group, 25 and 15 cases were respectively positive and negative by surgical results, and 23 and 17 cases were respectively positive and negative by needle biopsy (Tables-III and IV).

**Table-III:** Diagnostic Results of the Observation Group.

Surgical Results	Biopsy via 18F-FDG PET/CT Imaging Combined with 3D-Printed Templates		Total
	+	-	
+	32	1	33
-	1	6	7
Total	33	7	40

**Table-IV:** Diagnostic Results of the Control Group.

Surgical Results	Biopsy via 18F-FDG PET/CT Imaging		Total
	+	-	
+	19	6	25
-	4	11	15
Total	23	17	40

Compared with the control group, the observation group exhibited higher diagnostic accuracy and sensitivity and a lower missed diagnosis rate, with statistically significant differences (P < 0.05). However, no significant differences were observed in the specificity and misdiagnosis rate between the two groups (P > 0.05) (Table-V).

**Table-V:** Comparison of Diagnostic Efficacy between the two groups [n,(%)].

Group	Accuracy	Sensitivity	Specificity	Missed Diagnosis Rate	Misdiagnosis Rate
Observation	38(95.00)	32(80.00)	6(15.00)	1(2.50)	1(2.50)
Control	31(77.50)	19(47.50)	11(27.50)	6(15.00)	4(10.00)
*c*²	5.165	9.141	1.867	3.914	1.920
*P*	0.023	0.002	0.172	0.048	0.166

In the observation group: pneumothorax (n=1) and hemoptysis (n=1); in the control group: pneumothorax (n=1), hemorrhage (n=1), and hemoptysis (n=1). The incidence rate of complications in observation and control groups was 5.00% and 7.50%, respectively, with no significant differences (*χ*² = 0.213, P= 0.644).

## DISCUSSION

This study found that compared with ^18^F-FDG PET/CT imaging-guided biopsy alone, the application of ^18^F-FDG PET/CT imaging combined with 3D-printed templates for percutaneous aspiration biopsy of lung lesions results in fewer punctures, shorter puncture time, higher accuracy and sensitivity, and lower misdiagnosis rates (P<0.05). The analysis suggests that the high resolution of applying ^18^F-FDG PET/CT imaging combined with 3D-printed templates for percutaneous aspiration biopsy of the lung allows for accurate demonstration of lesion location, morphology, size, and relationships with surrounding tissues. This approach facilitates precise identification of the angle and depth of needle insertion during biopsy procedures and even ensures accurate positioning and operation for tiny lesions, with a high success rate of one-time biopsy to avoid repeated biopsies.^8^ 3D printing technology is utilized to create anatomical models based on medical imaging data, which can comprehensively demonstrate the characteristics and morphology of pulmonary tumors in patients and accurately locate nodules. The 3D-printed template, developed from this technology, is a specialized template for biopsy procedures, with minimal needle hole errors, excellent compatibility between the needle and the needle path, and a central cross-coordinate axis for precise positioning and adjustment of the needle position.^9^

Pulmonary occupying lesions, especially nodular lesions, often show no obvious clinical symptoms in the early stage due to their complex pathogenesis, making it challenging to determine the nature of lesions via imaging examinations, thus ultimately leading to patients often missing the optimal time for treatment when malignant lesions are diagnosed.^10^ Therefore, the key to improving the therapeutic effect and prognosis of pulmonary lesions lies in early screening, diagnosis, and treatment of the disease.^11^ Currently, there are various diagnostic methods for pulmonary lesions, including fiberoptic bronchoscopy and thoracoscopic lung biopsy. However, fiberoptic bronchoscopy has a low detection rate for tiny lesions and difficulty in accurately obtaining lesion tissues, while thoracoscopic lung biopsy is highly invasive and poorly tolerated by patients.^12^,^13^

In view of this, it is of great significance to select an effective detection method for patient prognosis. Research has confirmed that percutaneous lung biopsy is a safe and effective diagnostic procedure that accurately obtains tissue samples for pathological examination, thereby significantly improving the diagnosis accuracy of pulmonary lesions. CT-guided percutaneous aspiration biopsy of the lung is commonly used, which can clearly demonstrate the lesion and its relationship with surrounding structures, resulting in a high success rate of biopsy.^14^ However, it has a high false-negative biopsy rate, lacks real-time monitoring during the procedure, and requires multiple scans and adjustments of the needle location, as well as a high incidence of complications.^15^

The findings of this study suggested that the biopsy rate was 77.50% ~ 95.00% in the two groups. With organic integration of the functional imaging of PET with CT anatomical imaging, ^18^F-FDG PET/CT can effectively demonstrate the metabolic, proliferative, hypoxic, and apoptotic status of tumors, with significantly improved accuracy of needle biopsy for ^18^F-FDG hyper metabolic area.^16^ Relevant studies have shown an accuracy of 85.00%-100.00% for percutaneous lung biopsy guided by PET/CT imaging-infused lesion.^17^

Therefore, it is known that the application of ^18^F-FDG PET/CT imaging combined with 3D-printed templates for percutaneous aspiration biopsy of the lung enables precise localization of puncture, with a high success rate, safety, and significant diagnostic value.^18^ However, despite being minimally invasive, this is still an invasive procedure, making it impossible to completely avoid the occurrence of complications during the procedure.^19^,^20^

Therefore, attention should be paid to the following points should be noted during the procedure:

1. A comprehensive assessment of the patient should be conducted before biopsy, including vital signs, the size and location of tumors, and their relationship with surrounding tissues.
2. When a 3D-printed template is used for biopsy localization, the matching of the template with the tumor should be ensured to reduce the risk of biopsy failure.
3. Vital signs of patients should be closely monitored during the biopsy procedure, as well as changes in the biopsy site, so as to adjust the biopsy strategy promptly.
4. The patients should be followed up after biopsy to monitor the occurrence of complications and changes in the tumor.

### Limitations:

However, this was a single-center comparative retrospective cohort study, which came with limitations such as a small sample size and potential selection bias in the biopsy method. Therefore, multi-center prospective randomized controlled studies could be designed in the future to obtain more objective and scientific research results.

## CONCLUSIONS

As per findings of this study, ^18^F-FDG PET/CT imaging combined with 3D-printed templates for percutaneous aspiration biopsy of the lung has valuable clinical application in the diagnosis of pulmonary lesions, which may improve the success rate and accuracy of biopsy while reducing the number of biopsies. Moreover, it may provide more comprehensive tumor data for surgery and valuable evidence for clinical treatment, thereby making it a diagnostic and therapeutic method worthy of promotion.

### Authors’ Contributions:

**JZ** and **CR:** Carried out the studies, participated in collecting data, and drafted the manuscript, and are responsible and accountable for the accuracy or integrity of the work.

**JC, HS and XL: S**tatistical analysis, participated in its design and critical review.

All authors read and approved the final manuscript.

## References

[1] Jiao J, Li WW, Shang YH, Li XF, Jiao M (2023). Clinical effects of Chemotherapy combined with Immunotherapy in patients with advanced NSCLC and the effect on their nutritional status and immune function. Pak J Med Sci.

[2] Sung H, Ferlay J, Siegel RL, Laversanne M, Soerjomataram I, Jemal A (2021). Global Cancer Statistics 2020:GLOBOCAN Estimates of Incidence and Mortality Worldwide for 36 Cancers in 185 Countries. CA Cancer J Clin.

[3] Kuzucuoglu M, Gokyer A, Kula O, Yekdes AC, Sunal BS, Karamustafaoglu YA (2020). Relationship between the Size and Location of the Mass and Hilar and Mediastinal Lymph Node Metastasis in Early and Locally Advanced Non-Small Cell Lung Cancer. J Coll Physicians Surg Pak.

[4] Di Capua D, Bracken-Clarke D, Ronan K, Baird AM, Finn S (2021). The Liquid Biopsy for Lung Cancer:State of the Art, Limitations and Future Developments. Cancers (Basel).

[5] Sun XY, Chen TX, Chang C, Teng HH, Xie C, Ruan MM (2021). SUVmax of 18FDG PET/CT Predicts Histological Grade of Lung Adenocarcinoma. Acad Radiol.

[6] Tian Y, Chen C, Xu X, Wang J, Hou X, Li K (2021). A Review of 3D Printing in Dentistry:Technologies, Affecting Factors, and Applications. Scanning.

[7] Tongxin L, Jing X, Runyuan W, Wei W, Yu Z, Dong W (2022). Application Research of Three-Dimensional Printing Technology and Three-Dimensional Computed Tomography in Segmentectomy. Front Surg.

[8] Wadher K, Trivedi R, Wankhede N, Kale M, Umekar M (2021). 3D printing in pharmaceuticals:An emerging technology full of challenges. Ann Pharm Fr.

[9] Pardolesi A, Rolli L, Scanagatta P, Duranti L, Tavecchio L, Pastorino U (2021). Comparison of clinical and oncologic effectiveness between flexible 3-dimensional and bi-dimensional video-thoracoscopic surgery for lung cancer. Tumori.

[10] Barnett J, Belsey J, Tavare AN, Saini A, Patel A, Hayward M (2019). Pre-surgical lung biopsy in management of solitary pulmonary nodules:a cost effectiveness analysis. J Med Econ.

[11] Brückner M, Heblinski N, Henrich M (2019). Use of a novel vessel-sealing device for peripheral lung biopsy and lung lobectomy in a cadaveric model. J Small Anim Pract.

[12] Raza S, Chikarmane SA, Gombos EC, Georgian-Smith D, Frost EP (2018). Optimizing Success and Avoiding Mishaps in the Most Difficult Image-guided Breast Biopsies. Semin Ultrasound CT MR.

[13] Bingham BA, Huang SY, Chien PL, Ensor JE, Gupta S (2020). Pulmonary Hemorrhage Following Percutaneous Computed Tomography-Guided Lung Biopsy:Retrospective Review of Risk Factors, Including Aspirin Usage. Curr Probl Diagn Radiol.

[14] Zhang L, Ren Z, Xu C, Li Q, Chen J (2021). Influencing Factors and Prognostic Value of 18F-FDG PET/CT Metabolic and Volumetric Parameters in Non-Small Cell Lung Cancer. Int J Gen Med.

[15] Albano D, Gatta R, Marini M, Rodella C, Camoni L, Dondi F (2021). Role of 18F-FDG PET/CT Radiomics Features in the Differential Diagnosis of Solitary Pulmonary Nodules:Diagnostic Accuracy and Comparison between Two Different PET/CT Scanners. J Clin Med.

[16] Wu X, Huang Y, Zhao Q, Wang L, Song X, Li Y (2020). PD-L1 expression correlation with metabolic parameters of FDG PET/CT and clinicopathological characteristics in non-small cell lung cancer. EJNMMI Res.

[17] Cazzato RL, Garnon J, Shaygi B, Koch G, Tsoumakidou G, Caudrelier J (2018). PET/CT-guided interventions:Indications, advantages, disadvantages and the state of the art. Minim Invasive Ther Allied Technol.

[18] Russo A, De Miguel Perez D, Gunasekaran M, Scilla K, Lapidus R, Cooper B (2019). Liquid biopsy tracking of lung tumor evolutions over time. Expert Rev Mol Diagn.

[19] Niu Z, Chen K, Jin R, Zheng B, Gong X, Nie Q (2022). Three-dimensional computed tomography reconstruction in video-assisted thoracoscopic segmentectomy (DRIVATS):A prospective, multicenter randomized controlled trial. Front Surg.

[20] Tammam TF, Kamhawy GA (2019). Use of a curved needle to facilitate lateral sagittal infraclavicular block performance:a randomized clinical trial. J Anesth.

